# Supplementary material for: Fungicides, herbicides and bees: A systematic review of existing research and methods
Source: PLoS One. 2019 Dec 10;14(12):e0225743. doi: 10.1371/journal.pone.0225743 (PMC6903747; doi:10.1371/journal.pone.0225743)
Supplement: S1 Fig — (DOCX) [file pone.0225743.s001.docx]

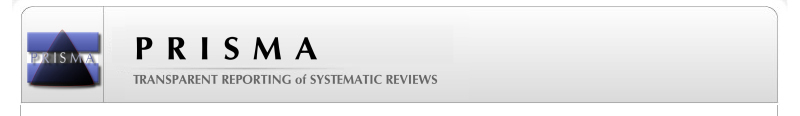
**PRISMA Flow Diagram**

Records excluded, no access to papers
(n =16)

Studies included in quantitative synthesis (meta-analysis)
(n = n/a)

Records screened
(n = 400)

Records after duplicates removed
(n = 400)

## Identification

## Eligibility

## Included

## Screening

Additional records identified through other sources
(n = 0)

Records identified through database searching
(n = 437)

Full-text articles assessed for eligibility
(n = 384)

Full-text articles excluded, with reasons – did not meet criteria
(n = 295)

*From:*  Moher D, Liberati A, Tetzlaff J, Altman DG, The PRISMA Group (2009). *P*referred *R*eporting *I*tems for *S*ystematic Reviews and *M*eta-*A*nalyses: The PRISMA Statement. PLoS Med 6(7): e1000097. doi:10.1371/journal.pmed1000097

**For more information, visit** [**www.prisma-statement.org**](http://www.consort-statement.org/)**.**

Studies included in qualitative synthesis
(n = 89)
