# Supplementary material for: Fungicides, herbicides and bees: A systematic review of existing research and methods
Source: PLoS One. 2019 Dec 10;14(12):e0225743. doi: 10.1371/journal.pone.0225743 (PMC6903747; doi:10.1371/journal.pone.0225743)
Supplement: S4 Table — (DOCX) [file pone.0225743.s005.docx]

**Fungicides, Herbicides and bees: A systematic review of existing knowledge**

Supplementary Information S4

Merissa Cullen^1*^, Linzi Thompson^2,3*^ , James C. Carolan^1^, Jane C. Stout^4^ & Dara A. Stanley^2,3,#^

^1^ Department of Biology, Maynooth University, Maynooth, Co. Kildare, Ireland

^2^ School of Agriculture and Food Science, University College Dublin, Belfield, Dublin 4, Ireland

^3^ Earth Institute, University College Dublin, Belfield, Dublin 4, Ireland

^4^ School of Natural Sciences, Trinity College Dublin, Dublin 2, Ireland

Table A. The top herbicide substance groups ranked from most studied to least.

| **Herbicide substance group** | **Number of studies alone and in combination with other pesticides or substances** |
| --- | --- |
| Phosophoneglycine | 15 |
| Triazine | 8 |
| Alkylchlorophenoxy | 5 |
| Bipyridylium | 5 |
| Chloroacetamide | 2 |
| Chlorophenoxy acid | 2 |
| Phenylurea | 2 |
| Triazinone | 2 |
| Urea | 2 |
| Aryloxyalkanoic acid | 1 |
| Benzothiazinone | 1 |
| Dinitroaniline | 1 |
| Inorganic herbicide | 1 |
| Pyridine compound | 1 |
| Sulfonylurea | 1 |
| Thiocarbamate | 1 |
| Triazolone | 1 |

Table B. The top fungicide substance groups ranked from most studied to least.

| **Fungicide substance group** | **Number of studies alone and in combination with other pesticides or substances** |
| --- | --- |
| Triazole | 30 |
| Strobilurin | 15 |
| Imidazole | 14 |
| Bendimidiazole | 13 |
| Chloronitrile | 13 |
| Phthalimide | 12 |
| Dicarboximide | 11 |
| Carboximide | 7 |
| Carbamate | 7 |
| Carboxamide | 6 |
| Anilinopyrimidine | 5 |
| Inorganic fungicide | 4 |
| Biofungicide | 3 |
| Phenylpyrrole | 3 |
| Unclassified fungicides | 3 |
| Chlorophenyl | 2 |
| Guanidine | 1 |
| Hydroxyanilide | 1 |
| Oxazole | 1 |
| Piperazine | 1 |
| Quinone | 1 |
| Sulphamide | 1 |
| Natural fungicide | 1 |
